# Supplementary material for: Reconciling Mining with the Conservation of Cave Biodiversity: A Quantitative Baseline to Help Establish Conservation Priorities
Source: PLoS One. 2016 Dec 20;11(12):e0168348. doi: 10.1371/journal.pone.0168348 (PMC5173368; doi:10.1371/journal.pone.0168348)
Supplement: S1 Dataset — (ZIP) [file pone.0168348.s002.zip › Taxa/Serra Norte/SN_2013/N4WS/Lista N4WS-65.pdf]

CAVIDADE N4WS-0065

Classe	Ordem	Fam/Outros	Gên/Outros	Espécie	S-U	U-S	Zon.
Arachnida	Acari	Ixodidae	*Amblyomma*	sp.1	X	X	E/P
Arachnida	Acari	Rhagidiidae		sp.1		X	P
Arachnida	Araneae	Araneidae		jovem	X		E
Arachnida	Araneae	Ctenidae		jovem	3		E/P
Arachnida	Araneae	Ochyroceratidae	*Ochyrocera*	sp.1	X	X	E/P
Arachnida	Araneae	Ochyroceratidae	*Speocera*	sp.1	X	X	P
Arachnida	Araneae	Ochyroceratidae	*Speocera*	sp.2	X		P
Arachnida	Araneae	Oonopidae	*Capitato*	sp.1	X		E
Arachnida	Araneae	Pholcidae		jovem		X	E
Arachnida	Araneae	Salticidae	*Freya*	sp.1		X	E
Arachnida	Araneae	Scytodidae	*Scytodes*	*eleonorae*		1	P
Arachnida	Araneae	Scytodidae	*Scytodes*	sp.1	1		E
Arachnida	Araneae	Theridiosomatidae	*Plato*	sp.1	X		P
Arachnida	Pseudoscorpiones	Chernetidae	*Spelaeochernes*	sp.1	X	X	P
Entognatha	Collembola	Isotomidae		sp.1	X		E
Entognatha	Collembola	Paronellidae		sp.1	X		E
Entognatha	Diplura	Campodeidae		sp.1		X	E/P
Insecta	Blattodea	Blaberidae		jovem	8	5	E/P
Insecta	Coleoptera	Carabidae		jovem	X		E/P
Insecta	Coleoptera	Staphylinidae		sp.7		X	E
Insecta	Coleoptera	Tenebrionidae		jovem	X	X	E
Insecta	Diptera	Culicidae	*Culex*	sp.	X		E
Insecta	Diptera	Psychodidae	*Sciopemyia*	*sordellii*	X	X	E/P
Insecta	Diptera	Tipulidae	Tipulinae	sp.	X		E
Insecta	Heteroptera	Reduviidae	Emesinae	jovem		1	E
Insecta	Heteroptera	Schizopteridae	Schizopterinae	sp.1	X	X	P
Insecta	Homoptera	Cixiidae		sp.4		X	E
Insecta	Hymenoptera	Formicidae	*Octostruma*	sp.1	X	X	E/P
Insecta	Hymenoptera	Ichneumonidae		sp.1	X		E
Insecta	Isoptera	Termitidae	*Nasutitermes*	sp.	X	X	E/P
Insecta	Lepidoptera	Noctuoidea		sp.2		X	E
Insecta	Lepidoptera	Tineoidea		sp.5	X		P
Insecta	Orthoptera	Phalangopsidae	*Phalangopsis*	sp.	15	13	P
Insecta	Psocoptera	Epipsocidae	*Epipsocus*	sp.2	X		E
Insecta	Psocoptera	Psyllipsocidae		jovem	X	X	P
Malacostraca	Isopoda	Dubioniscidae		sp.1		X	E
Amphibia	Anura	Leptodactylidae	*Leptodactylus*	cf. *vastus*	1		P
Amphibia	Anura	Strabomantidae	*Pristimantis*	cf. *fenestratus*	1	2	E/P
Amphibia	Anura			sp.4	1		P
